# Oral Shedding of an Oncogenic Virus Alters the Oral Microbiome in HIV+ Patients

**DOI:** 10.3389/fmicb.2022.882520

**Published:** 2022-04-19

**Authors:** Lu Dai, Yong-Chen Lu, Jungang Chen, Karlie Plaisance-Bonstaff, Shengyu Mu, J. Craig Forrest, Denise Whitby, Steven R. Post, Zhiqiang Qin

**Affiliations:** ^1^Department of Pathology, Winthrop P. Rockefeller Cancer Institute, University of Arkansas for Medical Sciences, Little Rock, AR, United States; ^2^Department of Medicine, Louisiana State University Health Sciences Center, Louisiana Cancer Research Center, New Orleans, LA, United States; ^3^Department of Pharmacology and Toxicology, University of Arkansas for Medical Sciences, Little Rock, AR, United States; ^4^Department of Microbiology and Immunology, Winthrop P. Rockefeller Cancer Institute, University of Arkansas for Medical Sciences, Little Rock, AR, United States; ^5^Viral Oncology Section, AIDS and Cancer Virus Program, Leidos Biomedical Research, Frederick National Laboratory for Cancer Research, Frederick, MD, United States

**Keywords:** KSHV, HIV, microbiome, oncogenic virus, oral microbiota

## Abstract

Kaposi’s Sarcoma (KS) caused by Kaposi’s sarcoma-associated herpesvirus (KSHV) continues to be the most common AIDS-associated tumor. Involvement of the oral cavity represents one of the most common clinical manifestations of this tumor. Numerous types of cancer are associated with the alterations of in components of the microbiome. However, little is known about how KSHV coinfection affects the oral microbiome in HIV+ patients, especially in a “pre-cancer” niche. Using 16S rRNA pyrosequencing, we found that oral shedding of KSHV correlated with altered oral microbiome signatures in HIV+ patients, including a reduction in the microbiota diversity, changing the relative composition of specific phyla and species, and regulating microbial functions. Furthermore, we found that *Streptococcus* sp., one of the most increased species in the oral cavity of HIV+/KSHV+ patients, induced KSHV lytic reactivation in primary oral cells. Together, these data indicate that oral shedding of KSHV may manipulate the oral microbiome to promote viral pathogenesis and tumorigenesis especially in immunocompromised patients.

## Introduction

Infection with Kaposi’s sarcoma-associated herpesvirus (KSHV) and the subsequent development of Kaposi’s sarcoma (KS) occur with a greater frequency following HIV infection or organ transplantation (Cesarman et al., 2019). KS consists of spindle cells (the tumor cell with endothelial derivatives), a proliferation of abnormal and leaky vessels and extravasated red blood cells with hemosiderin deposits (Cesarman et al., 2019). A prominent inflammatory infiltrate is also present early in the development of these lesions. Clinically, lesions have been described as patch, plaque, nodule, and tumor stages, with individual patients often displaying different types of lesions. AIDS-associated KS (AIDS-KS) can present as an aggressive disseminated disease affecting skin, lymph nodes, and visceral organs. Despite the reduced incidence of KS in the era of combined antiretroviral therapy (cART; Vanni et al., 2006), KS remains the most common AIDS-associated tumor and a leading cause of morbidity and mortality in this setting (Bonnet et al., 2004; Engels et al., 2008). Oral cavity involvement represents the initial manifestation of KS in 20%–60% of HIV-associated cases (Pak et al., 2005). The existing clinical data suggest that KSHV dissemination within and from the oral cavity are critical factors for KSHV infection and oral KS progression in HIV+ patients. Moreover, cART does not effectively reduce KSHV replication within the oropharynx or KSHV transmission (Miller et al., 2006).

Pathogenesis of periodontitis and associated chronic oral inflammation is dependent on the local microbiome within the gingival sulcus, and studies of the microbiota indicate that many of the same bacteria contributing to periodontitis in otherwise healthy persons also likely contribute to periodontitis for HIV+ patients. Several studies indicate a significantly higher prevalence of severe oral inflammation and periodontal disease for HIV+ patients, probably due to the change of microbial diversity over the course of HIV infection (Phiri et al., 2010; Mataftsi et al., 2011). Published data indicate that bacteria and viruses in the oral cavity interact to facilitate periodontal disease and that inflammatory factors accompanying periodontitis are associated with KS progression (Mesri et al., 2010; Slots, 2010). In addition, numerous metagenomics studies of the microbiome highlight microbial pattern modifications in various types of cancer and viral infections. In contrast, there are only limited data about the connection between infection with oncogenic viruses, such as KSHV, and microbiome signatures in HIV+ patients. One recent study showed a diminution of oral microbial diversity and enrichment of specific bacteria in HIV/KSHV co-infected individuals with oral KS (Gruffaz et al., 2020). However, all specimens in this study were collected from a cohort of HIV/KSHV co-infected individuals previously diagnosed with KS, while no KSHV negative HIV+ patients were involved and some of the patients currently had oral KS. Therefore, these data do not elucidate the “pre-cancer” niche of the oral cavity following KSHV infection. Thus, in the current study, we sought to understand whether KSHV oral shedding was able to alter oral microbiome in cohort HIV+ patients without KSHV-associated malignancies yet, which may further promote its pathogenesis and tumorigenesis.

## Materials and Methods

### Cell Culture, Bacteria, and Reagents

All of cell lines and bacteria strains were purchased from the American Type Culture Collection (ATCC) and cultured as recommended by the manufacturer. All experiments were carried out using cells harvested at low (<20) passages. All the chemicals if not indicated specifically were purchased from Sigma-Aldrich.

### Patients and Ethics Statement

The study was approved by the Institutional Review Boards for Human Research (IRB, No. 8079) at Louisiana State University Health Science Center (LSUHSC). All subjects were provided written informed consent. A total of 44 HIV+ patients with antiretroviral treatment (ART) in LSUHSC HIV Outpatient (HOP) Clinic were involved. There were 20 females and 24 males; the average age is 46.6 years (range 21–65 years).

### Plasma and Saliva Preparation

Whole blood was collected in heparin-coated tubes, and plasma was isolated by centrifugation. The KSHV infection status was determined by using quantitative ELISAs for identifying circulating IgG antibodies to KSHV proteins (LANA and K8.1; Mbisa et al., 2010; Benavente et al., 2011). To collect whole saliva, patients rinse with mouthwash, and saliva was collected in a wide-mouth 50 ml Nalgene tube. Typical volumes range between 3 and 5 ml of mouthwash. The patients were requested to not eat or smoke prior to providing the samples.

### Microbiome Sequencing and Analysis

The 16S rRNA V4 region from saliva samples was amplified and sequenced at the Argonne National Laboratory. The original sequencing data have been submitted to Gene Expression Omnibus (GEO) database (accession number: GSE190132). The sequencing data were mapped to SILVA reference database (release 132) and analyzed by the QIIME 2 software (version 2021.4; Quast et al., 2013; Bolyen et al., 2019). Data were visualized by QIIME 2, Microsoft Excel, or GraphPad Prism (version 9.3). For the prediction of microbial functions, the sequencing data were mapped to Greengenes reference database (version 13_5) and analyzed by QIIME2, prior to the final analysis by PICRUSt (version 1.1.4; Langille et al., 2013).

### qRT-PCR

Total RNA was isolated using the RNeasy Mini kit (Qiagen), and cDNA was synthesized using a SuperScript III First-Strand Synthesis SuperMix Kit (Invitrogen). Primers used for amplification of target genes were listed in Supplementary Table 1. Amplification was carried out using an iCycler IQ Real-Time PCR Detection System, and cycle threshold (Ct) values were tabulated in duplicate for each gene of interest in each experiment. “No template” (water) controls were used to ensure minimal background contamination. Using mean Ct values tabulated for each gene, and paired Ct values for *β-actin* as a loading control, fold changes for experimental groups relative to assigned controls were calculated using automated iQ5 2.0 software (Bio-Rad).

### Western Blot

Total cell lysates (20 μg) were resolved by 10% SDS-PAGE, transferred to nitrocellulose membranes, and immunoblotted with antibodies to phosphor (p)-p65 (Ser536)/total (t)-p65, p-p38 (Thr180/Tyr182)/t-p38, p-ERK (Thr202/Tyr204)/t-ERK, and Tubulin as loading controls (Cell Signaling). Immunoreactive bands were identified using an enhanced chemiluminescence reaction (Perkin-Elmer) and visualized by autoradiography.

### Plasmids Transfection

Cells were transfected with recombinant vectors of pcDNA-p65 or vector control using Lipofectamine™ 3000 reagent (Invitrogen). Transfection efficiency was normalized through co-transfection of a lacZ reporter construct and determination of β-galactosidase activity using a commercial β-galactosidase enzyme assay system (Promega).

### Statistical Analysis

Significant differences between experimental and control groups were determined using the two-tailed Student’s *t*-test.

## Results

### Signature of Oral Microbiome in HIV+ Patients With or Without KSHV Infection

In this study, whole blood and saliva specimens were collected from a cohort of 44 HIV+ patients as described previously (Dai et al., 2020). None of these patients reported previous KSHV-associated malignancies. The KSHV sero prevalence was determined using quantitative ELISA to identify circulating IgG antibodies to KSHV proteins (LANA and K8.1). KSHV oral shedding (viral copies) was determined by qPCR using specific primers designed for the viral major latent gene, *Lana (ORF73)*. Based on the ELISA and qPCR results, patients were divided into three groups: Group 1 (*n* = 25) patients with negative results for both ELISA and qPCR (Se-/Sa-) have no KSHV infection; Group 2 (*n* = 9) patients with positive results for ELISA but negative for qPCR (Se+/Sa-), and had or are having KSHV infection without oral virus shedding; and Group 3 (*n* = 10) patients with positive results for both ELISA and qPCR (Se+/Sa+) and have KSHV infection with virus oral shedding. There were no significant differences in age, gender, CD4 T cells count, and HIV viral loads among these groups (data not shown). Next, the hypervariable V4 region of the 16S rRNA gene present in saliva DNA was sequenced by next-generation sequencing at the Argonne National Laboratory. During the QC analysis, one sample from Groups 1 and 3 had low reads and were excluded from subsequent analysis.

**Figure 1:**
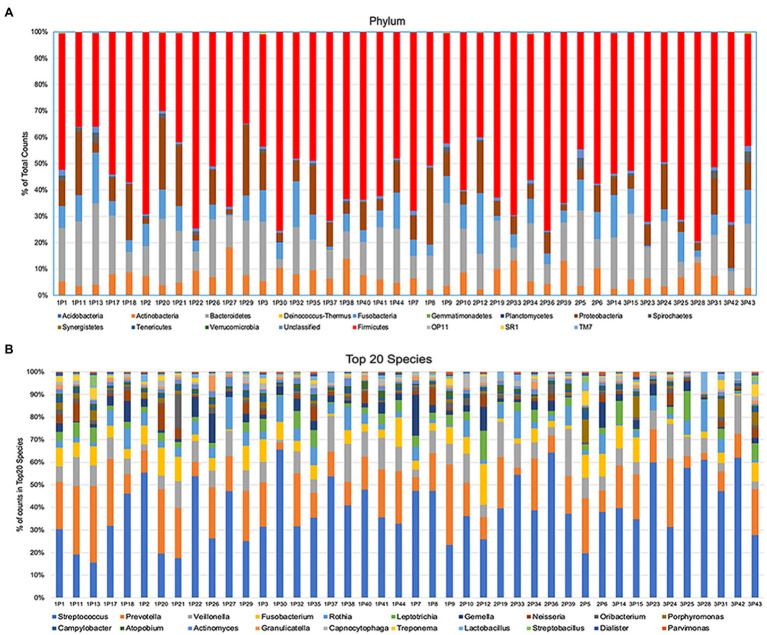
The components of microbiome at the phylum or species level from individual samples.

**Figure 2:**
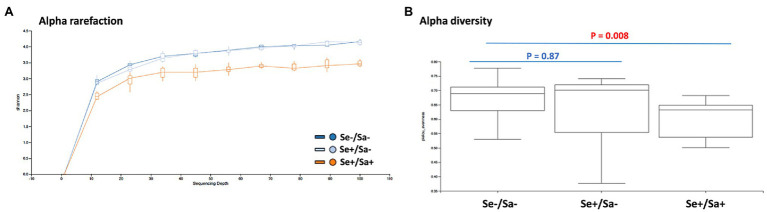
Diversity analysis of oral microbiome in HIV patients with or without Kaposi’s sarcoma-associated herpesvirus (KSHV) coinfection. **(A,B)** The Alpha rarefaction (Shannon index) and Alpha diversity (Pielou’s evenness) analysis of three groups of HIV patients with or without KSHV coinfection.

After alignment with QIIME database, unique representative sequences were classified into 1,366 operational taxonomic units (OTUs), from which 18 phyla, 28 classes, 59 orders, 119 families, 226 genera, and 374 species were identified. Differences between the relative abundances of OTUs and the taxonomic compositions of the bacterial populations at different taxonomic levels among the three patient groups were then determined. The patterns of phylum and species for each sample are shown in Figure 1. The Alpha rarefaction (Shannon index) and Alpha diversity (Pielou’s evenness) analyses both indicated a significant diminution of diversity and evenness in the Se+/Sa+ group (Group 3, *p* = 0.008) compared to the other two groups (Figures 2A,B).

### Oral Shedding of KSHV Alters Oral Microbiome Signatures in HIV+ Patients

**Figure 3:**
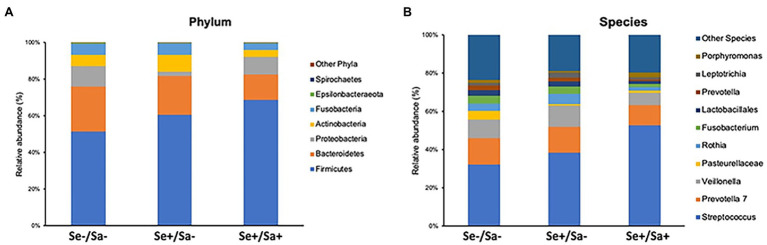
The top phylum and species in three groups of HIV patients with or without KSHV coinfection.

**Figure 4:**
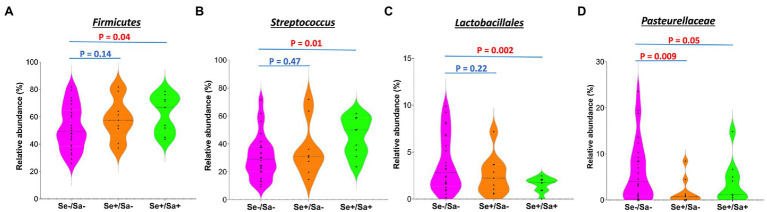
The significantly changed phyla or species in the group of HIV patients with KSHV oral shedding.

The top phylum and species for each group were shown in Figures 3A,B. When compared to the Se-/Sa- group (Group 1), *Firmicutes* and *Streptococcus* were significantly increased (*p* = 0.04 and 0.01, respectively) while *Lactobacillales* and *Pasteurellaceae* were significantly decreased (*p* = 0.002 and 0.05, respectively) in the Se+/Sa+ group; only *Pasteurellaceae* was significantly decreased (*p* = 0.009) in the Se+/Sa- group (Figures 4A–D). Interestingly, a previous study on oral KS (Gruffaz et al., 2020) also reported that *Firmicutes* was increased while *Lactobacillales* and *Pasteurellaceae* were decreased in saliva samples from oral KS patients with HIV infection. A heat map comparing additional species across the three groups is shown in Supplementary Figure 1. We then used the Phylogenetic Investigation of Communities by Reconstruction of Unobserved States (PICRUSt; Langille et al., 2013) to predict changes in oral microbiome functions. Interestingly, we found that several categories were changed in the Se+/Sa+ group (Group 3), including Bacterial motility proteins, glycerolipid metabolism, phosphotransferase system (PTS), *Staphylococcus aureus* infection, and two-component system (Supplementary Figure 2).

### Induction of KSHV Lytic Reactivation by *Streptococcus* Species Culture

**Figure 5:**
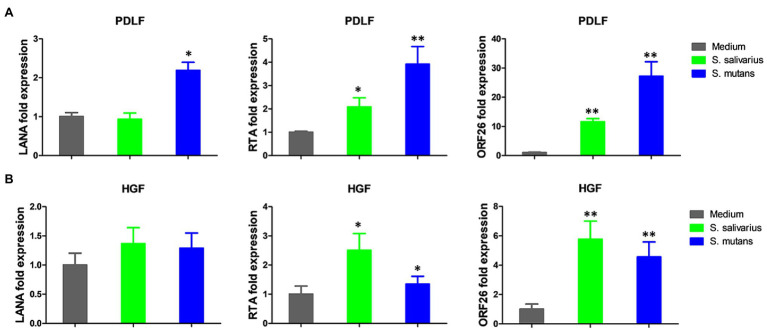
Induction of KSHV lytic gene expression by conditioned medium from *Streptococcus* species. **(A,B)** Oral fibroblasts periodontal ligament fibroblasts (PDLF) and human gingival fibroblasts (HGF) were infected with purified rKSHV.219 virus (MOI ~ 5) for 2 h. A 24 h later, cells were treated with filtered conditioned medium from *Streptococcus salivarius* C699 or *Streptococcus mutans* NCTC 10449 strain culture or fresh medium as a control (diluted as 1:50) for additional 48 h. Viral gene expression was measured and quantified using qRT-PCR. Error bars represent the S.D. for three independent experiments. *p < 0.05; **p < 0.01 (two-tailed Student’s *t*-test). PDLF, periodontal ligament fibroblasts; HGF, human gingival fibroblasts.

**Figure 6:**
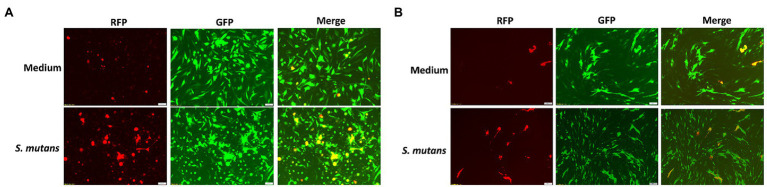
Induction of KSHV lytic reactivation by *Streptococcus* species. **(A,B)** Oral fibroblasts PDLF and HGF were infected with purified rKSHV.219 virus and treated with filtered conditioned medium of *Streptococcus* species as described above, and then images were captured by using fluorescence microscope.

Because *Streptococcus* is one of mostly increased species in the Se+/Sa+ group, we further explored its potential to impact KSHV pathogenesis. For our experiments, we used two common *Streptococcus* pathogenic species found in the oral cavity, *Streptococcus salivarius* and *Streptococcus mutans*. We found that treating latently infected primary human gingival fibroblasts (HGF) and periodontal ligament fibroblasts (PDLF) with filtered conditioned medium from either *S. salivarius* C699 or *S. mutans* NCTC 10449 strains induced significantly higher expression of KSHV lytic genes, especially *Rta* and *Orf26*, when compared to cells treated with fresh medium using qRT-PCR (Figures 5A,B). Immunofluorescence microscopy (RFP signal) further confirmed increased lytic reactivation induced by *Streptococcus* conditioned medium from virus-infected oral fibroblasts (Figures 6A,B).

### NF-κB Signaling Pathway Is Involved in *Streptococcus* Species Induced KSHV Lytic Reactivation

**Figure 7:**
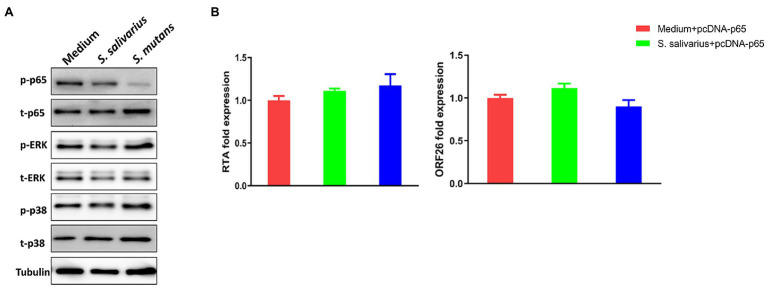
NF-κB signaling pathway is involved in *Streptococcus* species induced KSHV lytic reactivation. **(A)** PDLF and HGF were infected and treated as described above, and then protein expression was detected by using Western blot. **(B)** PDLF cells were infected first, then transfected with a recombinant construct encoding NF-κB p65 for 48 h, and treated with filtered conditioned medium from *Streptococcus* species culture for additional 48 h. Viral gene expression was quantified using qRT-PCR. Error bars represent the SD for three independent experiments.

Kaposi’s sarcoma-associated herpesvirus (KSHV) lytic reactivation is regulated by several intracellular signaling pathways such as MAPK and NF-κB (Broussard and Damania, 2020). We found that treatment of virus-infected oral fibroblasts with *Streptococcus* conditioned medium downregulated NF-κB activity (reduced phosphorylation of p65) but did not affect MAPK activity (Figure 7A). To determine if decreased NF-κB signaling is involved in viral lytic reactivation induced by *Streptococcus* conditioned medium, cells were transfected with a construct expressing NF-κB p65 (pcDNA-p65) or a vector control. *Streptococcus* conditioned medium-induced viral lytic gene expression (Figure 7B) was effectively blocked by transfecting with NF-kB p65, while the vector control had no effects (Supplementary Figure 3).

## Discussion

In the current study, we found that *Firmicutes* and *Streptococcus* were significantly increased while *Lactobacillales* and *Pasteurellaceae* were significantly decreased in oral cavity of HIV patients with KSHV oral shedding. Additional studies demonstrated higher abundances of *Streptococcus* in the salivary microbiome of several oral cancers or head and neck cancers, including salivary adenoid cystic carcinoma (SACC), oral squamous cell carcinoma (OSCC), and esophageal squamous cell carcinoma (ESCC; Jiang et al., 2021; Li et al., 2021; Stasiewicz and Karpinski, 2021). Thus, these data indicate potential contribution of *Streptococcus* to different oral cancers development. Indeed, our further assays showed that *Streptococcus* species culture effectively induced KSHV lytic reactivation from latently infected primary oral cells. This will not only facilitate virus dissemination in oral cavity, but also promote viral pathogenesis and oncogenesis, because recent findings indicate that some lytic proteins play a pivotal role in the initiation and progression of virus-associated cancers (Aneja and Yuan, 2017). For example, KSHV-encoded G-protein-coupled receptor (vGPCR) has been shown the ability to promote cell transformation and angiogenesis driving KS tumorigenesis.

Here, we predicted that KSHV oral shedding in HIV patients also changed several functional categories based on bioinformatics analysis, such as glycerolipid metabolism, phosphotransferase system (PTS), *Staphylococcus aureus* infection, and two-component system. In fact, glycerolipid metabolism and phosphotransferase system have been found related to the development of oral cancer, especially OSCC (Wang et al., 2018; Zhang et al., 2019). Interestingly, our previous studies showed that *S. aureus* and its components were able to increase KSHV entry and induce virus lytic reactivation (Dai et al., 2014, 2020). We also provided clinical evidence about the relevance of these two pathogens coinfection in oral cavities of cohort HIV+ patients. Interestingly, KSHV-infected oral cells showed greatly increased *S. aureus* internalization at early time points after bacterial infection (Dai et al., 2020). Two-component system (TCS) control proteins, harboring histidine kinase (HK) and response transcription regulator activities, have been uncovered in most bacteria and required for regulation of bacterial growth, virulence, or other important functions (Barrett and Hoch, 1998; Stephenson and Hoch, 2002). For example, *S. aureus* encodes 16 TCSs, which are required for bacterial survival and many other important functions so as potential drug targets. Therefore, our analysis raises a hypothesis that KSHV oral shedding may also promote some periodontal bacteria pathogenesis, change inflammatory microenvironment, increase periodontal diseases severity, and finally reach the win-win goal for these pathogens. Taken together, our experimental evidence further supports our findings from microbiota analysis, indicating that some antibacterial treatments (e.g., antibiotics, TCS inhibitors, and quorum sensing inhibitors) may interfere with bacteria-virus interaction to reduce virus reactivation and dissemination in oral cavity of patients. In future study, more functional assays (e.g., establishment of bacteria-virus coinfection system or adding specific bacterial productions and components into KSHV-infected cells culture) need to be performed for exploring the complicated interaction between this oncogenic virus and periodontal bacteria species.

## Data Availability Statement

The datasets presented in this study can be found in online repositories. The names of the repository/repositories and accession number(s) can be found in the article/**Supplementary Material**.

## Ethics Statement

The studies involving human participants were reviewed and approved by the Institutional Review Boards for Human Research at Louisiana State University Health Science Center (LSUHSC). The patients/participants provided their written informed consent to participate in this study.

## Author Contributions

ZQ: conception and design and study supervision. Y-CL and DW: development of methodology. LD, Y-CL, and JC: acquisition of data (provided animals, acquired and managed patients, provided facilities, etc.). LD, Y-CL, JC, SM, and ZQ: analysis and interpretation of data (e.g., statistical analysis, biostatistics, and computational analysis). LD, Y-CL, JF, DW, SP, and ZQ: writing, review, and/or revision of the manuscript. KP-B: administrative, technical, or material support (i.e., reporting or organizing data and constructing databases).

## Funding

This work was supported by NIH/NCI R01CA228166, the Arkansas Bioscience Institute, the major research component of the Arkansas Tobacco Settlement Proceeds Act of 2000 and in part with federal funds from the Frederick National Laboratory for Cancer Research, under contract number HHSN261201500003I and NCI contract 75N91019D00024. SM is supported by NIH R01 HL146713. Y-CL is supported by funding from the Winthrop P. Rockefeller Cancer Institute and Translational Research Institute KL2 Award (UL1 TR003107 and KL2 TR003108). Funding sources had no role in study design, data collection and analysis, decision to publish, or preparation of the manuscript.

## Conflict of Interest

The authors declare that the research was conducted in the absence of any commercial or financial relationships that could be construed as a potential conflict of interest.

## Publisher’s Note

All claims expressed in this article are solely those of the authors and do not necessarily represent those of their affiliated organizations, or those of the publisher, the editors and the reviewers. Any product that may be evaluated in this article, or claim that may be made by its manufacturer, is not guaranteed or endorsed by the publisher.

## Supplementary Material

The Supplementary Material for this article can be found online at: https://www.frontiersin.org/articles/10.3389/fmicb.2022.882520/full#supplementary-material
