# Supplementary material for: Oral Shedding of an Oncogenic Virus Alters the Oral Microbiome in HIV+ Patients
Source: Front Microbiol. 2022 Apr 19;13:882520. doi: 10.3389/fmicb.2022.882520 (PMC9063630; doi:10.3389/fmicb.2022.882520)

**Supplementary Table 1. Primer sequences used for qRT-PCR**

| Gene | Forward primers | Reverse primers |
| --- | --- | --- |
| LANA | 5'-TCCCTCTACACTAAACCCAATA-3' | 5'-TTGCTAATCTCGTTGTCCC-3' |
| RTA | 5’-CACAAAAATGGCGCAAGATGA-3’ | 5’-TGGTAGAGTTGGGCCTTCAGTT-3’ |
| ORF26 | 5’-GCTCGAATCCAACGGATTTG -3’ | 5’- AATAGCGTGCCCCAGTTGC-3’ |
| β-actin | 5’-ATCGTGCGTGACATTAAGGAG-3’ | 5’-GGAAGGAAGGCTGGAAGAGT-3’ |

**Supplementary figure legends**

**Supplementary Figure 1. The heatmap of Top 30 abundance species in 3 groups of HIV patients with or without KSHV coinfection.**

**Supplementary Figure 2. Functional gene families altered in the group of HIV patients with KSHV oral shedding.** The heatmap of functional gene families associated with microbiome was predicted by PICRUSt.

**Supplementary Figure 3. Transfection with control vectors cannot block KSHV lytic reactivation by conditioned medium from Streptococcus species.** PDLF cells were infected with purified KSHV virions (MOI~5), then transfected with control empty vectors (pcDNA) for 48 h, and treated with filtered conditioned medium from *Streptococcus* species culture (diluted as 1:50) for additional 48 h. Viral gene expression was quantified using qRT-PCR. Error bars represent the S.D. for 3 independent experiments. * = p<0.05; ** = p<0.01 (two-tailed Student's t-test).

**Supplementary Figure 1**


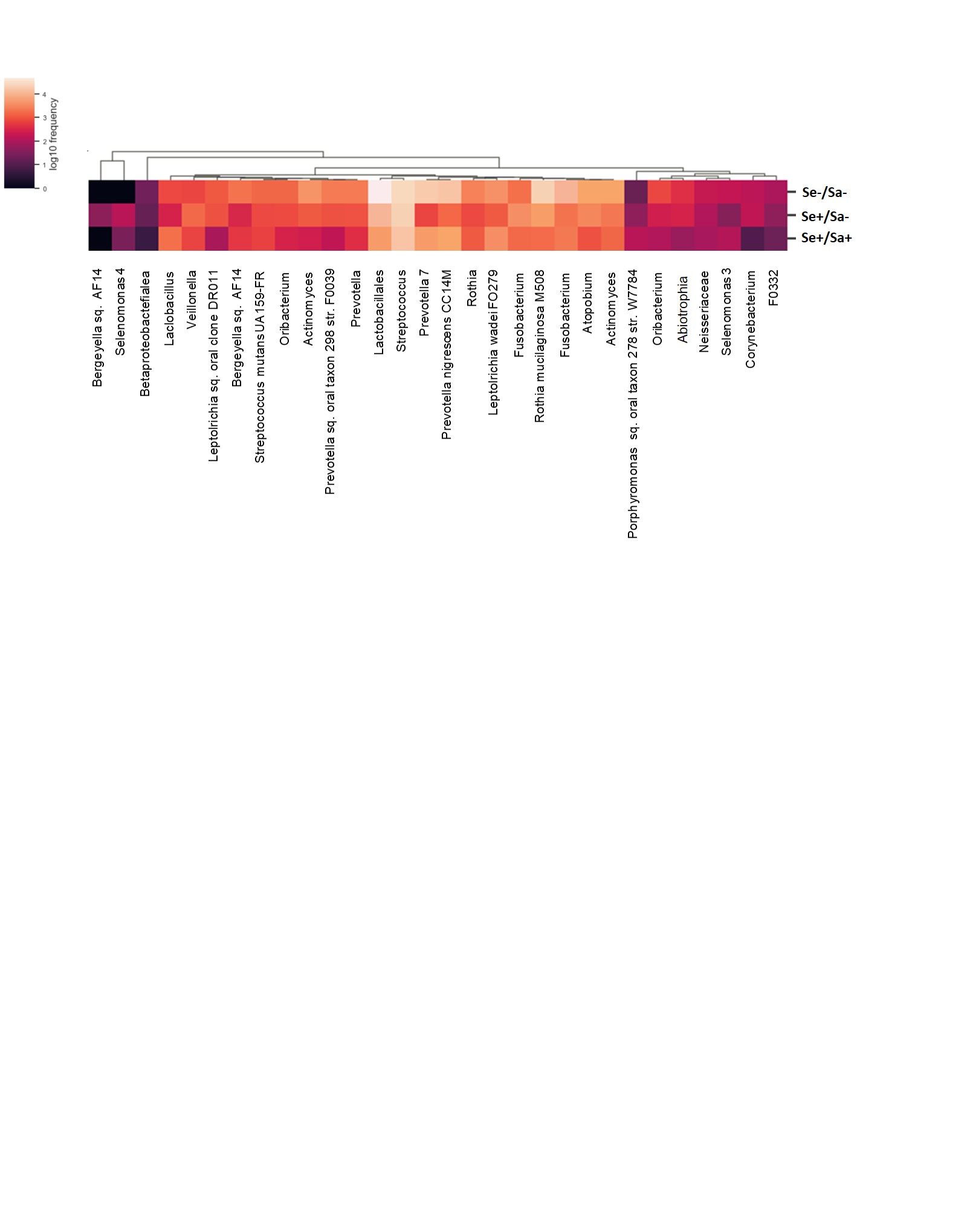


**Supplementary Figure 2**


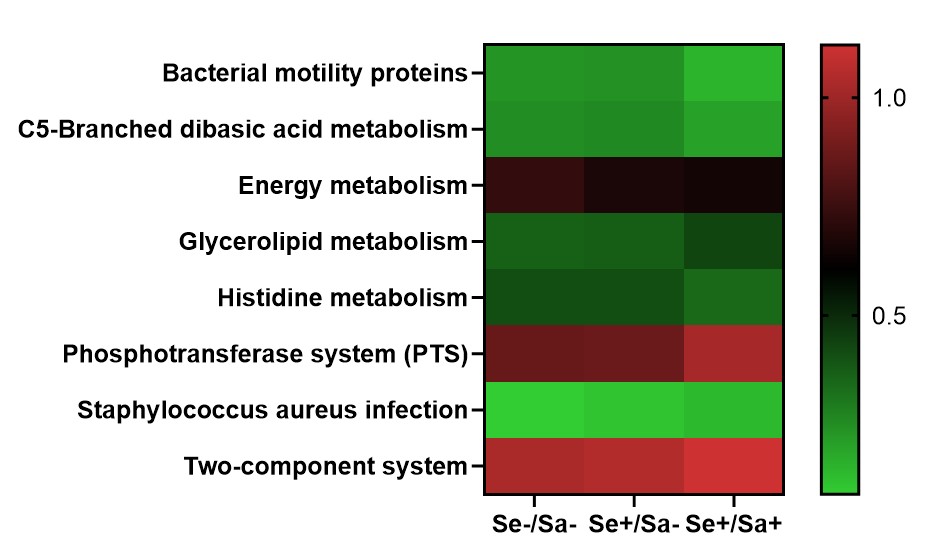


**Supplementary Figure 3**


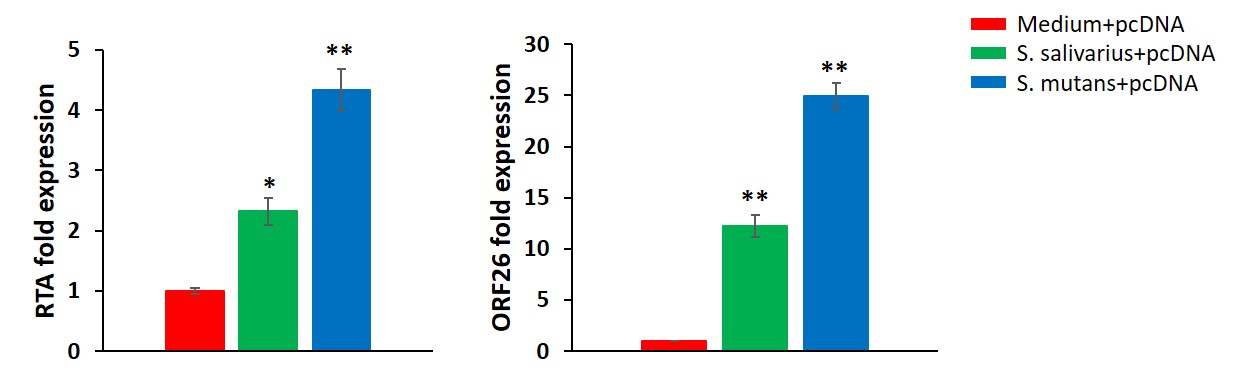

Supplement: Supplementary file 1 [file Data_Sheet_1.docx]
